# Amino acid classification based spectrum kernel fusion for protein subnuclear localization

**DOI:** 10.1186/1471-2105-11-S1-S17

**Published:** 2010-01-18

**Authors:** Suyu Mei, Wang Fei

**Affiliations:** 1 Shanghai Key Laboratory of Intelligent Information Processing, School of Computer Science, Fudan University, Shanghai, PR China

## Abstract

**Background:**

Prediction of protein localization in subnuclear organelles is more challenging than general protein subcelluar localization. There are only three computational models for protein subnuclear localization thus far, to the best of our knowledge. Two models were based on protein primary sequence only. The first model assumed homogeneous amino acid substitution pattern across all protein sequence residue sites and used BLOSUM62 to encode *k*-mer of protein sequence. Ensemble of SVM based on different *k*-mers drew the final conclusion, achieving 50% overall accuracy. The simplified assumption did not exploit protein sequence profile and ignored the fact of heterogeneous amino acid substitution patterns across sites. The second model derived the *PsePSSM *feature representation from protein sequence by simply averaging the profile PSSM and combined the *PseAA *feature representation to construct a kNN ensemble classifier *Nuc-PLoc*, achieving 67.4% overall accuracy. The two models based on protein primary sequence only both achieved relatively poor predictive performance. The third model required that GO annotations be available, thus restricting the model's applicability.

**Methods:**

In this paper, we only use the amino acid information of protein sequence without any other information to design a widely-applicable model for protein subnuclear localization. We use *K*-spectrum kernel to exploit the contextual information around an amino acid and the conserved motif information. Besides expanding window size, we adopt various amino acid classification approaches to capture diverse aspects of amino acid physiochemical properties. Each amino acid classification generates a series of spectrum kernels based on different window size. Thus, (I) window expansion can capture more contextual information and cover size-varying motifs; (II) various amino acid classifications can exploit multi-aspect biological information from the protein sequence. Finally, we combine all the spectrum kernels by simple addition into one single kernel called *SpectrumKernel+ *for protein subnuclear localization.

**Results:**

We conduct the performance evaluation experiments on two benchmark datasets: *Lei *and *Nuc-PLoc*. Experimental results show that *SpectrumKernel+ *achieves substantial performance improvement against the previous model *Nuc-PLoc*, with overall accuracy *83.47% *against *67.4%*; and *71.23% *against *50% *of *Lei SVM Ensemble*, against 66.50% of *Lei GO SVM Ensemble*.

**Conclusion:**

The method *SpectrumKernel*+ can exploit rich amino acid information of protein sequence by embedding into implicit size-varying motifs the multi-aspect amino acid physiochemical properties captured by amino acid classification approaches. The kernels derived from diverse amino acid classification approaches and different sizes of *k*-mer are summed together for data integration. Experiments show that the method *SpectrumKernel*+ significantly outperforms the existing models for protein subnuclear localization.

## Background

The cell nucleus is a highly complex organelle that controls cell reproduction, differentiation and regulates cell metabolic activities. Cell nucleus is subdivided into several sub-compartments, called subnuclear locations, where proteins are located to function properly. If mislocated, protein malfunction would cause cell disease. In-depth information about subcelluar localization may help a full understanding of genomic regulation and function. As compared to the general subcelluar localization, subcelluar localization is more challenging from biological viewpoints [1]. From computational viewpoints, the characteristic difference (e.g. amino acid composition, phylogenetic history, etc.) among the proteins in nucleus is far less distinct than that among proteins from different macro cell compartments, thus making it hard to achieve satisfactory predictive performance. Shen H et al. (2007) [2] derived the *PsePSSM *feature representation from protein sequence by simply averaging the profile PSSM and combined the *PseAA *feature representation to construct a *kNN *ensemble classifier *Nuc-PLoc*. *Nuc-PLoc *divided nucleus into 9 subnuclear locations and achieved 67.4% overall accuracy. Lei Z et al. (2005) [1] directly used BLOSUM62 to derive the similarity between the *k*-mers from two protein sequences, based on which an ensemble of SVM was constructed with different *k*-mers to draw the final conclusion. The model divided nucleus into 6 subnuclear locations and achieved 50% overall accuracy. To further boost the performance, Lei Z et al. (2007) [3] incorporated GO information into the SVM Ensemble classifier and achieved 66.5% overall accuracy. The unavailability of GO annotation would restrict the model's applicability. For novel proteins or proteins with many missing GO terms, the predictive performance would be rather poor, maybe still about 50%. We can see that the prediction for subnuclear localization is more difficult than general subcelluar localization.

Machine learning methods for predicting protein subcelluar location should take into account two major factors, one is to derive protein feature information and the other is to design predictive model. State-of-art feature extraction is data- and model-dependent. We should guarantee that the features not only capture rich biological information but also should be discriminative enough to construct a classifier for prediction. High throughout sequencing technique makes protein sequence cheaply available. In computational proteomics, many computational models are based on protein primary sequence. On the other hand, data integration becomes a popular method to integrate diverse biological data, including non-sequence information, such as GO annotation, protein-protein interaction, etc.

There are many models that extract features from protein sequence. Amino acid composition (AA) has close relation with protein subcelluar localization [4] and is the most frequently-used features, usually used together with other information for protein subcelluar localization [5,6]. Besides amino acid occurrence, pair-wise residue correlation and amino acid physiochemical properties are also incorporated to encode protein sequence, such as PseAA [7], Che-mAA [8], etc. Window-based *k*-mer histogram is another approach proposed to extract biological information from protein sequence, such as gapAA, dipeptide [6,8], and motif kernel [5,9], etc. AA is a special case for k-mer histogram when the window size equals 1. For k-mer histogram, the feature space expands exponentially with the window size k. To capture size-varying motif information and the context information around a specific amino acid residue, some approaches compress 20 amino acids into 7 groups according to amino acid physiochemical properties [10,11]. At both ends of a protein sequence, maybe there exists some sorting signal or anchoring signal for protein subcelluar localization. Hoglund A et al. (2006) [5] combined N-terminal signal, overall protein amino acid composition and eMotif information into a unified profile vector representation (PPV), and used the feature vector to construct a hierarchical SVM classifier for protein subcelluar localization. Schneider G et al. (2004) [12] gave a review on machine learning models using signal peptide for protein subcelluar location prediction as of 2004.

Protein phylogenetic information is another source for protein subcelluar localization. Edward M et al., (2000) [13] used Blast to generate a protein's profile distribution over several reference species, and revealed that proteins in the same subcelluar location manifest similar phylogenetic profile distribution, while proteins in different subcelluar locations were distinctly distributed. Several models extracted features from PSI-Blast profile such as PSSM and PSFM [14,15]. Mak M et al. (2008) [15] used PSI-Blast to generate the pro-file (PSSM & PSFM) for each query sequence, and derive a profile alignment kernel using dynamic programming to define two query sequences' similarity. Rangwala H et al. (2005) [16] used PSSM & PSFM to derive a string kernel for remote homology detection and fold recognition. The method calculated the profile similarity between all k-length fragments of consecutive amino acids to derive the similarity between two protein sequences, thus rather computationally intensive. Kuang R et al. (2005) [17] designed a profile kernel, a variant mismatch kernel [18], which allowed a *k *fragment to match its corresponding *k*-mer if the fragment fell within the positional mutation neighbourhood defined by *k*-mer self-entropy. Kuang R et al. (2009) [19] extended the profile kernel by simple kernel fusion for prediction of malaria degradomes. Besides profile information, domain is another source of evolutionary information that can be used for protein subcelluar localization. Richard M et al. (2002) [20] analyzed the domain co-occurrence pattern of eukaryotic proteins and found that proteins in the same subcelluar location have similar domain co-occurrence pattern. Some other researches used flat binary domain vector to represent protein [21]. In such a sparse high-dimensional representation, the information about domain content and partition boundary is discarded. Mei S et al. (2009) [22] proposed a multiple instance learning model to make use of the domain boundary information along protein sequence, where domain is regarded as an instance and the protein sequence is regarded as a bag. Ensemble learning is a commonly-adopted data integration method used to integrate heterogeneous data, such as GO annotation [23,24], PPI network [19], etc. A little differently, Lee K et al. (2008) [8] concatenated the feature vectors from different data sources. The great challenge in those models is how to objectively estimate the model performance and how to predict a novel protein when neither GO annotation nor protein-protein interaction would be available. The model estimation was conducted only in the optimistic scenario that both training set and test set had GO or PPI information available. The published model performance may be overestimated. On the other hand, when GO or PPI information is unavailable, some base classifiers of the ensemble classifier would fail to work and may contribute nothing to novel protein prediction. So, it is worth discussing whether ensemble learning is fit for heterogeneous data integration.

However, kernel method can be used to fuse the heterogeneous information (GO/PPI information, etc.) by kernel matrix summation, with 0 filling the matrix for missing information. The expensive information can be used to tune SVM parameters, so that the knowledge contained in the expensively-acquired data can be transferred to the cheap data and the expensive information is not necessary for novel protein prediction. Kernel method has witnessed successful applications in computational biology in recent years [15-19,25], where *k*-mer based kernels [16-19,25] can be seen as variant spectrum kernel and mismatch kernel that incorporated protein sequence profile information. *K*-mer feature representation can capture the contextual information around an amino acid residue and cover conserved motifs. Alexander Z et al. [9] combined amino acid composition kernel and motif kernel using Multiple Kernel Learning (MKL) to automatically optimize the weights of kernel matrices. The optimal weights were derived using Semi-Infinite programming instead of convex Semi-definite programming to accelerate computation at the sacrifice of global optimum.

In this paper, we only use the amino acid information of protein sequence without any other information to design a widely-applicable model for protein subnuclear localization. We use K-spectrum kernel to exploit the contextual information around an amino acid and the conserved motif information. Besides expanding window size, we adopt various amino acid classification approaches to capture diverse aspects of amino acid physiochemical properties. Each amino acid classification generates a series of spectrum kernels based on different window size. Thus, (I) window expansion can capture more contextual information and cover size-varying motifs; (II) various amino acid classifications can exploit multi-aspect biological information from the protein sequence; (III) amino acid classification approaches can compress 20 amino acids to a certain content, so as to allow larger window size and reduce the dimensionality of feature space. Finally, we combine all the spectrum kernels by simple addition into one single kernel called *SpectrumKernel+ *for protein subnuclear localization.

## Methods

### Spectrum kernel

Kernel method [26,27] maps data points into possibly high-dimensional feature space, where a linear hyperplane can be optimized using quadratic convex programming to separate two-class data with maximum margin. Assume mapping function Φ(*x*), the computation of the inner product <Φ(*x*_*i*_), Φ(*x*_*j*_) > in the high-dimensional feature space can be implemented in the original space using kernel trick, *K *(*x*_*i*_, *x*_*j*_) = < Φ (*x*_*i*_), (Φ*x*_*j*_), > such that no explicit mapping function or even explicit feature representation is required. We need only the similarity between two data points to derive the semi-definite kernel function. Many kernel functions have been derived to measure the similarity between two protein sequences. Leslie, C. et al. (2002) [25] defined a spectrum kernel function that computed the similarity between the *k*-spectrum of two protein sequences. *K*-spectrum is the set of all *k*-length consecutive sub-sequences (*k*-mer). Given a protein sequence *x*, amino acid set Σ(|Σ| = *l*), we define a feature map *X *→ , Φ(*x*) = , where *ϕ*_*a *_(*x*) = number of occurrences of *a *in *x*, thus *k*-spectrum kernel is defined as *K*_*k *_(*x*, *y*) = < Φ (*x*), (Φ*y*)>. Assume each *k*-mer is indexed as *kmer*_(*i*)_, *i *= 1, 2, ..., *length *(*x*) - *k *+1 by the position *i *where the *k*-mer sliding window is located, we can see that *kmer*_(*i*) _contributes 1 to *ϕ*_*a *_(*x*) (i.e. *ϕ*_*a*_(*x*) = *ϕ*_*a*_(*x*) + 1) where *a *= *kmer*_(*i*)_, *a *∈ Σ^*k*^. Then, spectrum kernel is defined as *SpectrumKernel*_*k*_(*x*, *y*) = < Φ (*x*), Φ(*y*) >, where Φ(*x*) is sequence-to-feature mapping function. Here, we use Gaussian kernel instead:

### Amino acid classification based spectrum kernel fusion

*K*-spectrum kernel can capture the contextual information around an amino acid residue and the *k*-mer occurrence patterns can reveal some conserved sub-sequences (e.g. motif). To capture more contextual information and cover a variety of size-varying motifs, we expand the window size to generate a series of *SpectrumKernel*_*k *_(*k *= 1, 2, ...). Since the feature space expands exponentially with window size |Σ|^*k*^, we should set upper limit for window size *k *for computational sake. On the other hand, 20 amino acids may seem redundant from a particular aspect of physiochemical properties (e.g. polarity), thus we can compress 20 amino acids into several groupings according to a certain criteria of amino acid classification. Thus, we can further expand the window size for compressed amino acid set but also can exploit different aspects of amino acid properties. According to polarity and charge, amino acids can be divided into 4 categories (4-cat); According to the density-functional theory method B3LYP/6-31G and molecular modelling approach [11], we can derive 7 categories (7-cat). Other amino acid classification methods *ms*, *lesk*, *F-Ic4 *are taken from [28] (see Table 1). The window limit for each amino acid classification method also is given in Table 1. It should be noted that the original *k*-spectrum kernel used 20 amino acids without adopting other amino acid classification approaches.

**Table 1:** Amino acid classification

*Method*	*Window limit*	*Amino acid classification*								
***4-Cat***	6	ALVIFWMP	STYCNGQ	KRH	DE	RK	DE	C		
***7-Cat***	4	AGV	ILFP	YMTS	HNQW					
***20-Cat***	3	A G V I L F P Y M T S H N Q W R K D E C								
***ms***	4	AVLIMC	WYHF	TQSN	RK	ED	GP			
***lesk***	4	AST	CVILWYMPF	HQN	RK	ED	G			
***F-Ic4***	4	AWM	GST	HPY	CVIFL	DNQ	ER	K		
***F-Ic2***	3	AWM	GS	HPY	CVI FL	DNQ	ER	K	T	
***F-IIIc4***	3	ACV	HPL	DQ	S	ERGN	F	IMT	KW	Y
***F-Vc4***	3	AWHC	G	LEPV	KYMT	IN	Q	D	S	

Only one state-of-art k-mer histogram may not be enough to extract biological information from protein sequence and construct a discriminative classifier. We combine all the SpectrumKernels based on different window size and different amino acid classification methods. When combining multiple kernels, the optimal weight vector *w *= (*w*_1_, *w*_2_, ..., *w*_*n*_) should be automatically derived from data. *K *= *w*_1_*K*_1 _+ *w*_2_*K*_2 _+ ... + *w*_*n*_*K*_*n*_, when *K*_*i *_≥ 0, *i *= 1, 2..., *n*, semi-definite programming can be applied (Lanckriet G et al. 2004) [29]; otherwise, semi-indefinite programming (Alexander Zien et al. 2007) [9] can be used to derive the optimal *w*. Both methods have rather large complexity. Here, we use simple weight vector *w*_*i *_= 1, *i *= 1, 2.., *n*, with the assumption that all feature representations have equal significance. Thus, we define *SpectrumKernel+ *as follows:

## Results

### Dataset description

We choose *Nuc-PLoc *[2] and *Lei *benchmark datasets to evaluate *SpectrumKernel+ *performance. The *Nuc-PLoc *dataset is collected from the Swiss-Prot database (version 52.0 released on 6 May 2007) [30] and divides cell nucleus into 9 subnuclear locations and the number of proteins in the locations is unbalanced, the largest *Nucleolus *has 307 proteins and the smallest *Nuclear PML body *has only 13 proteins. The dataset has total 714 proteins. The *Lei *benchmark dataset [1] is collected from Nuclear Protein Database [31], chiefly from human and mouse, and divides cell nucleus into 6 subnuclear locations and totals up to 504 proteins. This dataset is also unbalanced.

### Model evaluation and experimental setting

*Nuc-PLoc *[2] and *Lei *[1] used leave-one-out cross validation (LOOCV) to estimate model performance. For simple classifier like *kNN*, the training is not so time-consuming and LOOCV may be acceptable for small dataset in such a case. For complex model, LOOCV may take unendurable long time to train and predict. 5-fold cross validation is a commonly-accepted model evaluation approach in computational biology, so we use 5-fold cross validation instead to evaluate *SpectrumKernel+ *performance. For 5-fold cross validation, the protein dataset is randomly split into five disjoint parts with equal size. The last part may have 1-4 more examples than the former 4 parts in order for each example to be evaluated on the model. One part of the dataset is used as test set and the remained parts are jointly used as training set. The procedure iterates for five times, and each time a different part is chosen as test set. We use four commonly-adopted measures: Sensitivity (SE), Specificity (SP), Matthew's correlation coefficient (MCC) and Overall Accuracy. MCC is often used to evaluate the balance of model prediction. LIBSVM http://www.csie.ntu.edu.tw/~cjlin/libsvm/ is used together with *SpectrumKernel+*, with the parameter setting "-s 0 -t 4 -c 1000 -e 0.0001".

### Comparison with baseline model

The performance comparison between *SpectrumKernel+ *and thebaseline models is illustrated in Table 2 & Table 3 respectively, where better results are highlighted in bold and the winner is underlined.

**Table 2:** Performance comparison on 714 *Nuc-PLoc *subnuclear protein dataset

*Subnuclear location*	*Size*	*Nuc-PLoc*	*SpectrumKernel+*I		
		
		MCC	SP	SE	MCC
***Chromatin***	99	0.60	0.7131	**0.8788**	0.7573
***Heterochromatin***	22	0.52	0.6364	0.3182	0.4386
***Nuclear envelope***	61	0.53	**0.8689**	**0.8689**	**0.8569**
***Nuclear matrix***	29	0.52	0.3750	0.3103	0.3171
***Nuclear pore complex***	79	0.70	**0.9367**	**0.9367**	**0.9290**
***Nuclear speckle***	67	0.43	0.7606	**0.8060**	0.7608
***Nucleolus***	307	0.57	**0.9231**	**0.9772**	**0.9133**
***Nucleoplasm***	37	0.31	0.7857	0.2973	0.4688
***Nuclear PML body***	13	0.32	0.7143	0.3846	0.5181
***Overall Accuracy***		67.40%		**84.03%**	

**Table 3:** Performance comparison on 504 *Lei *subnuclear protein dataset

*Subnuclear location*	*size*	*Lei SVM ensemble*		*SpectrumKernel+*I			*SpectrumKernel+*II		
		
		SE	MCC	SP	SE	MCC	SP	SE	MCC
***PML Body***	38	0.2900	0.1720	0.2093	0.2368	0.1630	0.1111	0.1053	0.0463
***Nuclear Lamina***	55	0.4360	0.3380	0.4167	0.4545	0.3718	0.5185	0.5091	0.4611
***Nuclear Speckles***	56	0.3570	0.3630	0.8611	0.5536	0.6636	**0.8667**	0.6964	0.7539
***Chromatin***	61	0.1970	0.2600	0.4643	0.4262	0.3813	0.6429	0.5902	0.5703
***Nucleoplasm***	75	0.2270	0.2060	0.4500	0.4800	0.3834	0.5256	0.5467	0.4649
***Nucleolus***	219	0.7670	0.3670	0.8603	0.8995	0.7992	**0.8979**	**0.9635**	**0.8795**
***Overall Accuracy***		50.00%		64.29%			**71.23%**		

The experiment on *Nuc-PLoc *dataset adopts the amino acid classification set {Cat-4, cat-7, cat-20, ms, lesk, F-IC4}, referred to as *SpectrumKernel+*I. As shown in Table 2, *SpectrumKernel+*I performs much better than *Nuc-PLoc*, with overall accuracy 84.03% against 67.40%. The measure MCC reveals that *SpectrumKernel+*I also achieves better performance on most subnuclear locations, except *Heterochromatin *and *Nuclear matrix. Nuc-PLoc *did not give the results of measure SP and SE. According to the measures SP and SE, we can see that *SpectrumKernel+*I achieves satisfactory predictive performance on large-data subcelluar locations: *chromatin, nuclear envelope, nuclear pore complex, nuclear speckle *and *nucleolus*. The largest-data *nucleolus *has less misclassification from and to other locations (SP: 0.9231; SE: 0.9772; MCC: 0.9133). On small-data subnuclear locations: *Heterochromatin, Nuclear matrix, Nucleoplasm *and *Nuclear PML body, SpectrumKernel+*I achieves rather poor performance, whereas *Nuc-PLoc *performed even worse. Maybe it is much less training data that causes the poor performance. As to the second benchmark dataset, we first conduct experiment using the amino acid classification set {Cat-4, cat-7, cat-20, ms, lesk, F-IC4}. *SpectrumKernel+*I achieves overall accuracy *64.29%*, much higher than *50% *of *Lei SVM Ensemble *[1]. See Table 3 for details. To verify the assumption that more information about amino acid classification may further increase accuracy, we add three additional amino acid classification approaches: *F-Ic2, F-IIIc4, F-Vc4*, thus we further evaluate *SpectrumKernel+ *using the expanded amino acid classification set {Cat-4, cat-7, cat-20, ms, lesk, F-IC4, F-Ic2, F-IIIc4, F-Vc4}, called *SpectrumKernel+*II. We can see from Table 3 that *SpectrumKernel+*II achieves 71.23% overall accuracy against 64.29% of *SpectrumKernel+*I with increase 6.94%, and against 50.00% of *Lei SVM Ensemble *with remarkable 21.23%. *SpectrumKernel+*II performs far better than *Lei SVM Ensemble *on all subnuclear locations except *PML Body*. The three additional amino acid classification approaches surely improve the performance in terms of both overall accuracy and all subnuclear locations according to the measures: SP, SE and MCC.

*SpectrumKernel+*I contains 25 spectrum kernels and *SpectrumKernel+*II contains 34 spectrum kernels, far less than 65 kernels combined in [9]. Here, we don't compare *SpectrumKernel+*II with *Lei GO SVM ensemble *[3], which achieved *66.50% *overall accuracy. The reason is that GO information will restrict the model's application, when GO information is missing for those proteins to be predicted, *Lei GO SVM ensemble *would degrade to the sequence-based *Lei SVM ensemble. SpectrumKernel+*II &*SpectrumKernel+*I are based on the amino acid information of protein sequence only.

### Comparison with individual spectrum kernel

**Figure 1:**
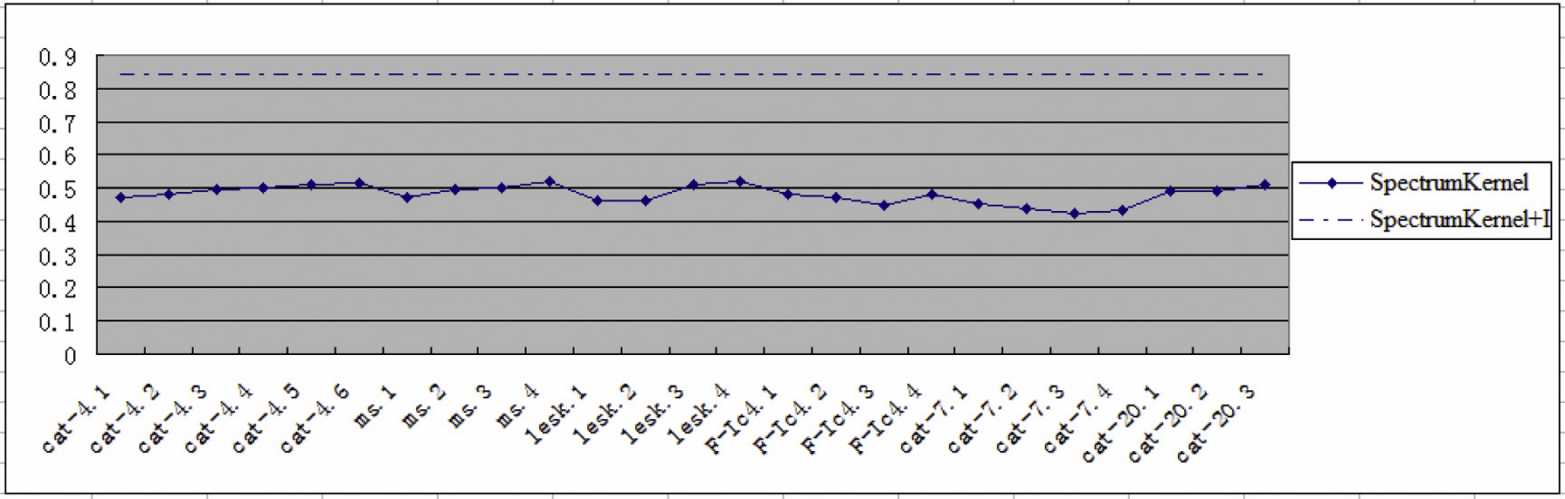
**Performance comparison between individual *SpectrumKernel *and *SpectrumKernel+*I on *Nuc-PLoc *dataset**.

To validate the effectiveness of kernel fusion, we evaluate the performance of all individual kernels generated by different amino acid classifications and different window sizes on the same 5-fold cross validation training & test sets. As shown in Figure 1, the x-axis x1.x2 denotes amino acid classification (x1) and window size (x2). From Figure 1, the accuracy of individual *SpectrumKernels *ranges between 42.58% and 51.96%. *Cat-20.3; cat-4.4; cat-4.5; cat-4.6; ms.4; lesk.3 *and *lesk.4 *capture more information; *F-Ic4 *second; *Cat-7 *the worst. However, the kernel fusion *SpectrumKernel+I *increases predictive accuracy steeply to 84.03%, with accuracy increase against individual spectrum kernels between 32.07% and 41.45%.

**Figure 2:**
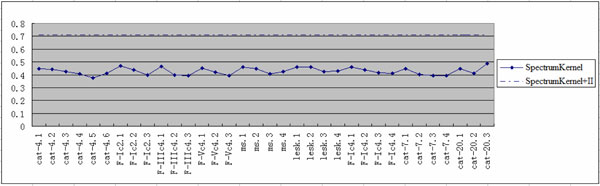
**Performance comparison between individual *SpectrumKernel *and *SpectrumKernel+*IIon *Lei *dataset**.

In Figure 2, three additional amino acid classifications: *F-Ic2, F-IIIc4, F-Vc4 *are added. The accuracy of individual spectrum kernel ranges between *37.50% *and *48.61%*, whereas the kernel fusion *SpectrumKernel+*II increases accuracy to *71.23*%, with accuracy increase between *22.62% *and *33.73%*. The result reveals that kernel fusion can combine multiple-aspect information of protein sequence to sharply increase the predictive accuracy.

## Discussion

This paper proposes a kernel method called *SpectrumKernel+ *that defines diverse spectrum kernel functions on the basis of different amino acid classification approaches and different window sizes. Different amino acid classification can capture different aspect of amino acid physiochemical properties, while varying window size can capture more contextual information and cover size-varying motifs. Therefore, *SpectrumKernel+ *can exploit diverse amino acid information from the protein sequence. *SpectrumKernel+ *has an obvious advantage that only the amino acid information of the protein sequence is required for protein subnuclear localization, without GO annotation, PSI-Blast profile, etc. Kernel fusion by using expensive information such as GO annotation, protein-protein interaction, etc. to tune SVM parameters may be a more graceful design than *Lei*'s GO SVM ensemble, because parameters tuning can transfer expensive information to the model trained on cheap data, and the expensive information is allowed missing when predicting a novel protein. In addition, this paper first explicitly introduces various amino acid classification approaches for spectrum kernel design, to the best of our knowledge, which is useful to extract rich information from the protein sequence for data integration. Experiments show that *SpectrumKernel+ *steeply increases the predictive accuracy as compared against the single-aspect spectrum kernel.

Actually, it may further improve *SpectrumKernel+'s *performance by adding more amino acid classification information and using Multiple Kernel Learning to optimally weigh the derived kernel matrices.

## Conclusion

Amino acid classification not only implicitly captures a certain aspect of amino acid physiochemical property, but also greatly reduces the dimensionality of *k*-mer feature space, allowing the model to cover longer motifs. Multi-aspect amino acid properties are embedded into the *k*-mer patterns (motif) by combining amino acid classification with spectrum kernel, which provides a novel analysis of protein sequence. Combining all the derived kernels helps integrate multi-aspect information of protein sequence and boost the performance of predictive model.

## Competing interests

The authors declare that they have no competing interests.

## Authors' contributions

MSY conducted the computational modelling. WF conceived and supervised the study. All authors read and approved the final manuscript.

## References

[B1] Lei Z Dai Y An SVM-based system for predicting protein subnuclear localizations BMC Bioinformatics 2005 6 291 10.1186/1471-2105-6-291 16336650 PMC1325059

[B2] Shen H Chou K Nuc-PLoc: a new web-server for predicting protein subnuclear localization by fusing PseAA composition and PsePSSM Protein Eng Des Sel 2007 20 561 567 10.1093/protein/gzm057 17993650

[B3] Lei Z Dai Y Assessing protein similarity with Gene Ontology and its use in subnuclear localization prediction BMC Bioinformatics 2006 7 491 10.1186/1471-2105-7-491 17090318 PMC1660555

[B4] Cedano J Aloy P P'erez-Pons J Querol E Relation between amino acid composition and cellular location of proteins Journal of Molecular Biology 1997 266 594 600 10.1006/jmbi.1996.0804 9067612

[B5] Hoglund A Donnes P Blum T Adolph H Kohlbacher O MultiLoc: prediction of protein subcellular localization using N-terminal targeting sequences, sequence motifs and amino acid composition Bioinformatics 2006 22 10 1158 1165 10.1093/bioinformatics/btl002 16428265

[B6] Bhasin M Raghava G ELSpred: SVM-based method for subcellular localization of eukaryotic proteins using dipeptide composition and PSI-BLAST Nucleic Acid Res 2004 32 Web Server W414 W419 10.1093/nar/gkh350 15215421 PMC441488

[B7] Chou K Prediction of protein subcellular locations by incorporating quasi-sequence-order effect Biochemical and Biophysical Research Communications 2000 278 477 483 10.1006/bbrc.2000.3815 11097861

[B8] Lee K Chuang H Beyer A Sung M Huh W Lee B Ideker T Protein networks markedly improve prediction of subcellular localization in multiple eukaryotic species Nucleic Acids Research 2008 36 20 e136 10.1093/nar/gkn619 18836191 PMC2582614

[B9] Alexander Z Cheng S An. Automated combination of kernels for predicting protein subcellular localization NIPS workshop on Machine Learning in Computational Biology 2007

[B10] Dijk A Bosch D Braak C Krol A Ham R Predicting sub-Golgi localization of type II membrane proteins Bioinformatics 2008 24 16 1779 1786 10.1093/bioinformatics/btn309 18562268 PMC7110242

[B11] Shen J Zhang J Luo X Zhu W Yu K Chen K Li Y Jiang H Predicting protein-protein interactions based only on sequences information PNAS 2007 104 11 4337 4341 10.1073/pnas.0607879104 17360525 PMC1838603

[B12] Schneider G Fechner U Review advances in the prediction of protein targeting signals Proteomics 2004 4 1571 1580 10.1002/pmic.200300786 15174127

[B13] Edward M Ioannis X Alexander M David E Localizing proteins in the cell from their phylogenetic profiles Proc Natl Acad Sci USA 2000 97 12115 12120 10.1073/pnas.220399497 11035803 PMC17303

[B14] Guo J Lin Y TSSub: eukaryotic protein subcellular localization by extracting features from profiles Bioinformatics 2006 22 14 1784 1785 10.1093/bioinformatics/btl180 16787975

[B15] Mak M Guo J Kung S PairProSVM: protein subcellular localization based on local pairwise profile alignment and SVM IEEE/ACM Transactions on Computational Biology and Bioinformatics 2008 5 3 416 422 10.1109/TCBB.2007.70256 18670044

[B16] Rangwala H Karypis G Profile-based direct kernels for remote homology detection and fold recognition Bioinformatics 2005 21 23 4239 4247 10.1093/bioinformatics/bti687 16188929

[B17] Kuang R Ie E Wang K Siddiqi M Freund Y Leslie C Profile-based string kernels for remote homology detection and motif extraction J Bioinform Comput Biol 2005 3 527 550 10.1142/S021972000500120X 16108083

[B18] Leslie C Eskin E Cohen A Weston J Noble W Mismatch string kernels for discriminative protein classification Bioinformatics 2004 20 4 467 476 10.1093/bioinformatics/btg431 14990442

[B19] Kuang R Jianying Gu Hong Cai Yufeng Wang Improved prediction of malaria degradomes by supervised learning with SVM and profile kernel Genetica 2009 136 189 209 10.1007/s10709-008-9336-9 19057851 PMC2721224

[B20] Richard M Jörg S Peer B Chris P Predicting protein cellular localization using a domain projection method Genome Research 2002 12 1168 1174 10.1101/gr.96802 12176924 PMC186639

[B21] Jia P Qian Z Zeng Z Cai Y Li Y Prediction of subcellular protein localization based on functional domain composition Biochemical and Biophysical Research Communications 2007 357 366 370 10.1016/j.bbrc.2007.03.139 17428441

[B22] Mei S Wang F Structural domain based multiple instance learning for predicting bacteria Gram-positive protein subcelluar location International Joint Conferences on Bioinformatics, Systems Biology and Intelligent Computing 2009

[B23] Chou K Shen H Cell-PLoc: A package of web-servers for predicting subcellular localization of proteins in various organisms Nature Protocols 2008 3 153 162 10.1038/nprot.2007.494 18274516

[B24] Tung T Lee D A method to improve protein subcellular localization prediction by integrating various biological data sources BMC Bioinformatics 2009 10 Suppl 1 S43 10.1186/1471-2105-10-S1-S43 19208145 PMC2648781

[B25] Leslie C Eskin E Noble W The spectrum kernel: a string kernel for SVM protein classification Proc Pac Biocomput Symp 2002 7 566 575 11928508

[B26] Taylor J Cristianini N Kernel Methods for Pattern Analysis 2004 Cambridge University Press

[B27] Vapnik V Statistical Learning Theory 1998 Springer 10.1109/72.788640 18252602

[B28] Alejandro S Ernesto P Segovia L Protein homology detection and fold inference through multiple alignment entropy profiles Proteins 2008 70 248 256 10.1002/prot.21506 17671981

[B29] Lanckriet G DeBie T Cristianini N Jordan M Noble W A statistical framework for genomic data fusion Bioinformatics 2004 20 16 2626 2635 10.1093/bioinformatics/bth294 15130933

[B30] Boeckmann B Bairoch A Apweiler R Blatter M Estreicher A Gasteiger E Martin M Michoud K Donovan C Phan I The SWISS-PROT protein knowledgebase and its Supplement TrEMBL Nucleic Acids Research 2003 31 365 370 10.1093/nar/gkg095 12520024 PMC165542

[B31] Dellaire G Farrall R Bickmore W The Nuclear Protein Database (NPD): subnuclear localisation and functional annotation of the nuclear proteome Nucl Acids Res 2003 31 328 330 10.1093/nar/gkg018 12520015 PMC165465

